# Virtual thoracoscopic imaging for accurate pulmonary nodule localization: clinical experience

**DOI:** 10.1007/s00595-024-02945-4

**Published:** 2024-10-16

**Authors:** Yuka Kadomatsu, Megumi Nakao, Shoji Okado, Harushi Ueno, Taketo Kato, Shota Nakamura, Toyofumi Fengshi Chen-Yoshikawa

**Affiliations:** 1 Department of Thoracic Surgery, Nagoya University Graduate School of Medicine, 65 Tsurumai-Cho, Showa-Ku, Nagoya, 466-8550 Japan; 2 Graduate School of Medicine, Human Health Sciences, Kyoto University, Kyoto, Japan

**Keywords:** Computed tomography, Pulmonary nodule, Video-assisted thoracoscopic surgery

## Abstract

The increasing detection of small pulmonary nodules on computed tomography (CT) warrants simple and effective nodule localization methods. We describe our clinical experience using an experimental computer that displays virtual thoracoscopic images. This device constructs three-dimensional images from preoperative CT scans and simulates the deflated lung parenchyma in the lateral decubitus position. Five patients underwent lung resection using this technology. The device provided images that closely resembled actual thoracoscopic images in all cases. This method addresses the limitations of other localization techniques such as allergic reactions and mechanical marker-related complications. The method only requires preoperative CT images, and the process is semi-automatically performed by specifying the nodule location, thoracoscopic camera insertion site, and camera angle. This study is still in the preliminary phase and has several limitations. However, this method has the potential to accurately predict nodule locations and eliminate the many risks associated with other techniques.

**Supplementary Information:**

The online version contains supplementary material available at 10.1007/s00595-024-02945-4.

## Technology

The increasing detection of small pulmonary nodules on computed tomography (CT) warrants simple and effective nodule localization methods. Various localization techniques have been reported; however, each method has inherent risks, mandating case-by-case selection of the most optimal approach [1]. Several localization methods have reported success rates from the high 80 s to the mid-90 s [1]. These targeted nodules are expected to be early stage lung cancers or benign diseases, and the preferred approach includes minimally invasive surgeries, such as video-assisted thoracoscopic surgery (VATS) or robotic-assisted thoracic surgery (RATS). Under the thoracoscopic view, image-based assessment becomes paramount for accurately determining the nodule’s location and the clue of each marking method.

We used three localization techniques at our institution: CT-guided hookwire placement, virtual-assisted lung mapping (VAL–MAP) technique [2], and intraoperative cone beam CT [3]. Similarly, we achieved success in determining small nodules using these approaches; however, instances of unsuccessful localization, necessitating larger anatomical resection inclusive of the nodule, were encountered.

Preoperative CT-guided reconstruction techniques for surgical resection have evolved in recent years. Many technologies have been developed for static structures, including mediastinal structures and bones [4]. In the case of lung parenchyma, dynamic deformations caused by respiration are a barrier to three-dimensional (3D) reconstruction techniques.

Previously, we reported a deformable lung simulation application called resection process map (RPM) and used it in clinical practice [5, 6]. The details of the RPM theory have been reported previously [7]. Briefly, RPM is an original application that demonstrates deformable lung 3D images followed by preoperative lung CT. We herein describe our clinical experience with small-nodule identification using this surface deformation technique and virtual thoracoscopic images in conjunction with conventional methods.

## Patients and methods

Five patients underwent lung resection with these virtual thoracoscopic images from August 2023 to May 2024. The institutional review board of Nagoya University School of Medicine approved this study on March 8, 2016 (2015–0458) and all patients provided their written informed consent before the day of surgery. Table 1 shows the preoperative marking methods and targeted nodule data. At our institution, decisions regarding the marking of small nodules, including the selection of the marking method, were made during the preoperative conference. In each case, an attempt was made to palpate the nodule. Contingency plans for unclear nodule localization during surgery were established in advance. Depending on the specific details of the patient’s case, the decision to either not resect or proceed with a more extensive anatomical resection was made in advance. This protocol has been consistently used in the application of this new technique. In the five cases included in this study, the nodules were successfully resected.

**Table 1:** Preoperative marking methods and targeted tumor characteristics

	Original marking	Location	Approach	Procedure	Nodule diameter
Case 1	VAL–MAP	Rt S9	VATS	wedge	7 mm
Case 2	VAL–MAP	Rt S1	VATS	wedge	18 mm
Case 3	hook wire	Rt S9	RATS	wedge	6 mm
Case 4	VAL–MAP	Rt S1	RATS	segmentectomy	17 mm
Case 5	None	Rt S6	VATS	wedge	15 mm

*RATS* robotic-assisted thoracic surgery, *Rt*, right, *VAL*–*MAP* virtual-assisted lung mapping, *VATS* video-assisted thoracoscopic surgery

## Technique

Fujifilm Medical Co. Ltd. provided an experimental computer with the function of evaluating lung deformation change and virtual thoracoscopic images. This experimental equipment can model the deflated lung parenchyma, considering the lateral decubitus position during surgery, in addition to the conventional function of constructing 3D images from preoperative CT scans. The degree of deflation is adjusted to any level using a slider. Virtual thoracoscopic images are displayed by specifying the thoracoscope insertion point and the target nodule position on the CT image, along with the viewing angle. In addition, changes in the field of view due to the depth of the thoracoscope are freely expressed (Supplementary Material 1).

## Clinical experience

CT is routinely performed after the marking methods in the case of VAL–MAP and CT-guided marking. Preoperatively, additional marking points and lung surfaces where the target nodule is located are designated on the experimental computer, and the predicted nodules in the lateral position corresponding to the intraoperative situation are shown after the deflation process.

## Case 1: right lateral basal segment (S9) with VATS wedge resection

**Fig. 1:**
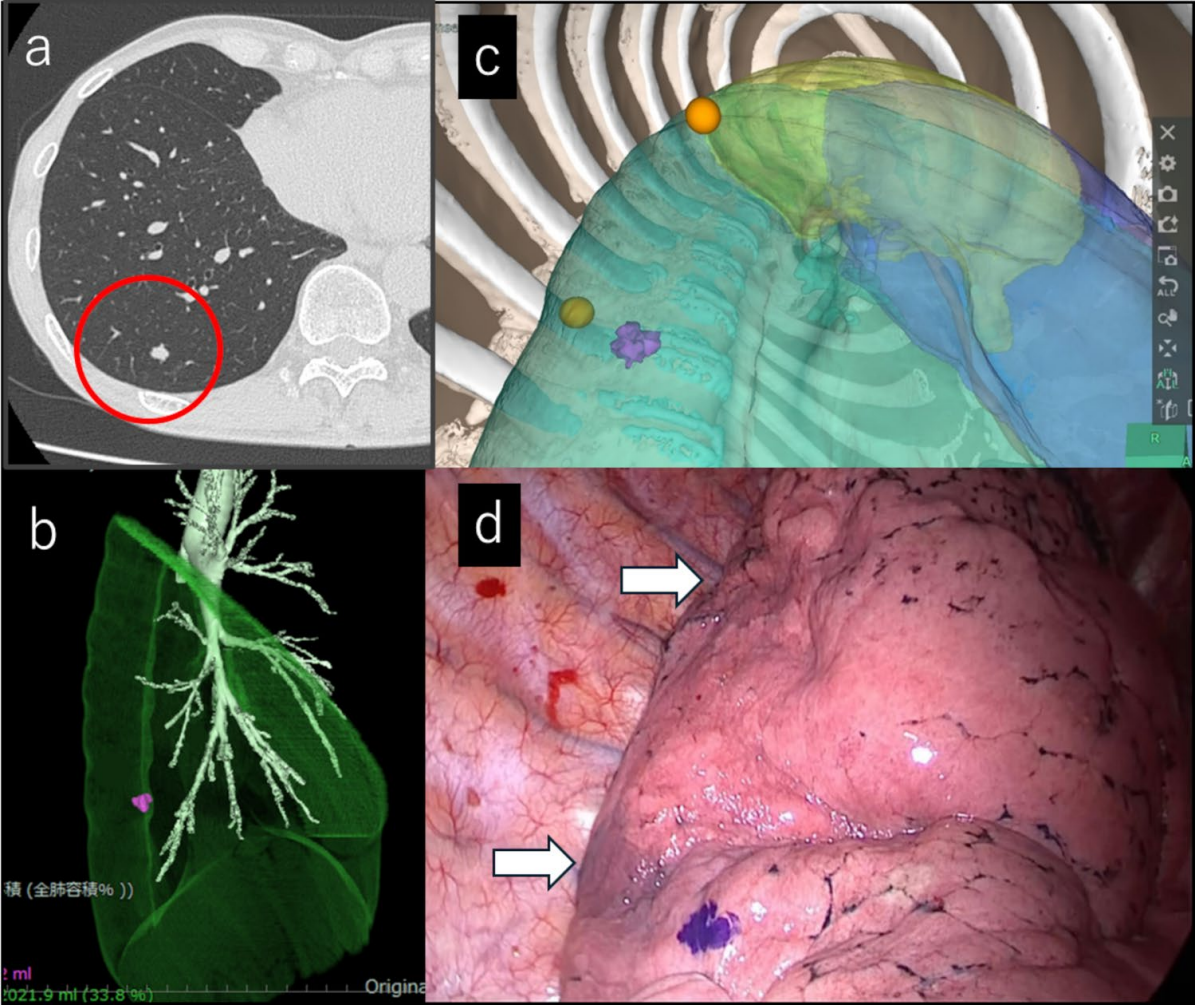
**a** Preoperative CT image showing a 7-mm nodule on the right S9. **b** Synapse Vincent image created from preoperative CT. **c** Virtual thoracoscopic images obtained preoperatively. The yellow sphere indicates the VAL–MAP marking location and the purple denotes the nodule position. **d** Intraoperative thoracoscopic image. The white arrow indicates the VAL–MAP dye. The dye-stained site represents the surgeon’s predicted nodule location, based on the marking positions and nodule location on the laboratory equipment

We report the case of a metastatic lung tumor located in the right S9 segment with a maximum diameter of 7 mm (Fig. 1a). VATS and VAL–MAP were planned because of the anticipated difficulty of visualizing the nodule. SYNAPSE VINCENT (Fujifilm, Tokyo, Japan) was used to illustrate the position in 3D (Fig. 1b). Two additional markings were placed using VAL–MAP to encompass the nodule. The experimental computer displayed additional marking points and a target nodule (Fig. 1c). An intraoperative image is shown with the VAL–MAP marking sites, indicated by orange arrows (Fig. 1d). The dye-stained site indicated the surgeon’s predicted location of the nodule based on the marking positions and nodule location on the laboratory equipment. Wedge resection was performed thoracoscopically around the marked area, confirming the inclusion of the targeted nodule (Supplementary Material 2).

## Case 4: right apical segment (S1) with RATS segmentectomy

**Fig. 2:**
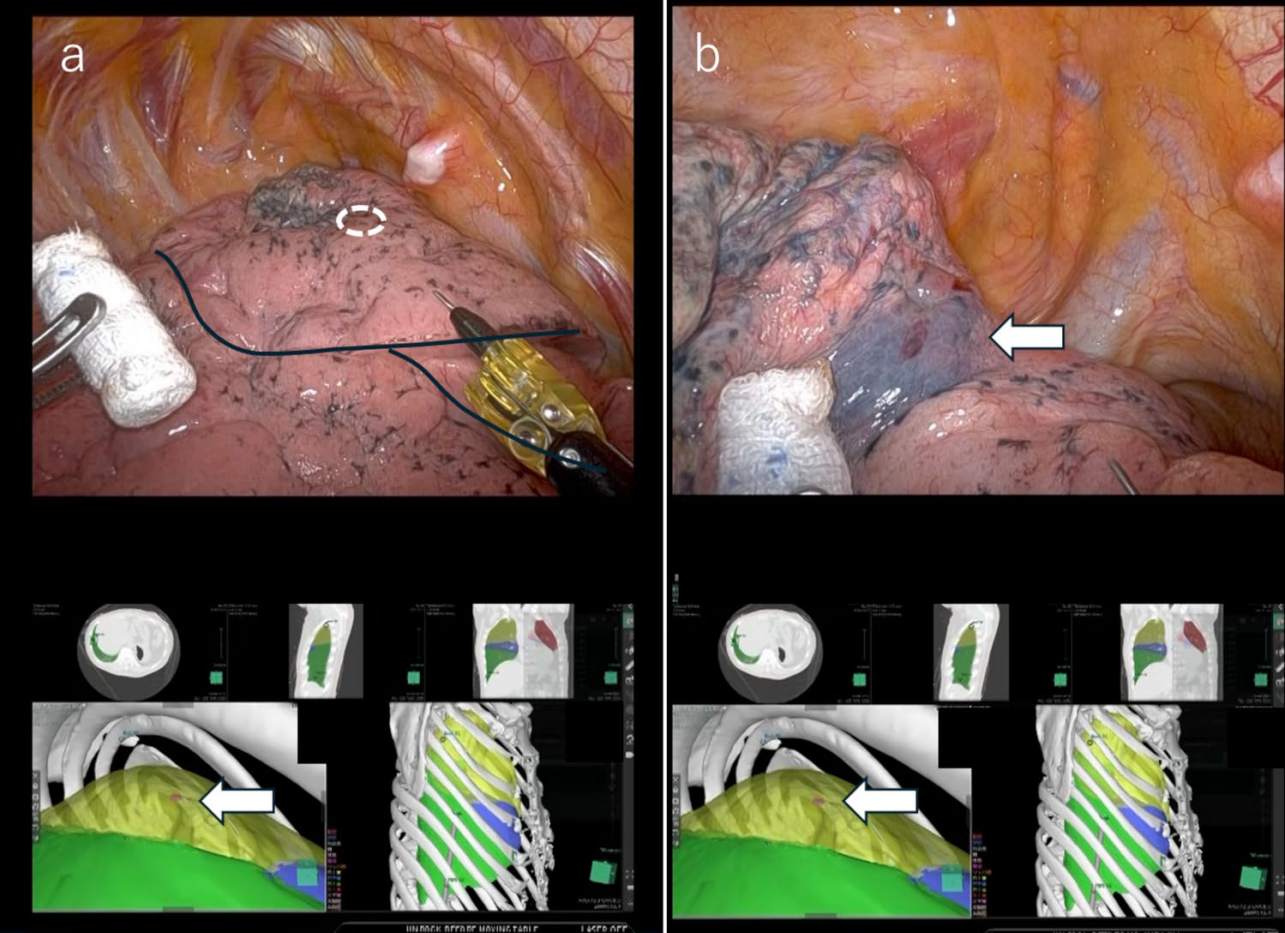
**a** The intraoperative console screen during robotic surgery. The white dashed circle indicates the estimated tumor location. The tumor is located on the mediastinal side; thus, it cannot be visualized by simply deflating the lung. The black solid lines indicate the boundaries of each lobe. The lower part of the screen displays the virtual thoracoscopic image output by the Tile Pro function, with the white arrow demonstrating the nodule’s location. **b** An intraoperative image of the mobilized lung parenchyma. The white arrow indicates dye marking from VAL–MAP, demonstrating the nodule’s location

A 17-mm diameter tumor (part-solid nodule) was located on the mediastinal side of the right S1. Right S1 segmentectomy was planned, and preoperative marking was conducted to ensure a sufficient tumor margin. A single VAL–MAP marking was made at approximately the same location as the tumor. Intraoperatively, the Da Vinci TilePro function (Intuitive Surgical) was used to display images from the experimental device on the console screen, allowing for a more precise comparison between the location of the nodule and the real thoracoscopic image. Retracting the lung parenchyma anteriorly revealed the dye at a similar location, as indicated by the virtual thoracoscope because the nodule and dye were located on the mediastinal side of the upper lobe. Figure 2 shows the operative console images.

## Comment

Intraoperatively, we considered multiple factors that changed the tumor location, including the deflated lung and the transition to a lateral decubitus position, along with images obtained via an oblique thoracoscope. This approach allowed us to obtain preoperative predicted images that closely matched the intraoperative thoracoscopic images. This study reviewed five cases, including four cases using a combination of VAL–MAP and hookwire, and one case with no preoperative marking. The experimental device provided images that closely resembled the actual thoracoscopic images from preoperative CT data in all cases.

Currently, various preoperative marking methods are becoming more widely used to ensure confirmed small-nodule resection. Methods using agents raise concerns about agent-related allergic reactions, and mechanical marking methods such as hookwires carry a risk of complications. This method eliminated the possibility of side effects and complications. The only required material is preoperative CT images, and the process on the experimental device involves simply specifying the location of the nodule, insertion site of the thoracoscopic camera, and camera angle. Image generation was then performed semi-automatically. Creating predictive thoracoscopic images for the left side is equally possible, although the target nodule in this study was accidentally limited to the right side. Nodules located on the mediastinal side, which are difficult to reach using CT-guided marking, did not pose a problem for this technique, as demonstrated in case 4. Overall, the primary advantage of this technique is its simplicity as it does not require specialized skills. Furthermore, it is not dependent on a skilled technician or on the location of the tumor to be identified. This method can also be used in conjunction with existing physical markers because it is non-invasive.

This study is still in the preliminary phase and has several limitations. First, the thoracoscopic port positions must be predetermined on the experimental device, indicating that any changes to port positions during actual surgery or observations from multiple port positions require virtual thoracoscopic image reconstruction. Second, our previously reported RPM technology enabled matching lung deformation through mouse manipulation, but the current experimental device can only display a simple deflated lung state and cannot track manual manipulation-related deformations. Finally, “virtual thoracoscopic imaging” can potentially enhance the precision of tumor localization. However, the accuracy of this technique remains in the validation phase. Furthermore, it is intended for use in conjunction with approved small-nodule marking methods or palpation.

## Supplementary Information

Below is the link to the electronic supplementary material.

Supplementary file1 (MP4 14143 KB)

Supplementary file2 (MP4 30531 KB)

## Data Availability

An experimental computer for displaying virtual thoracoscopic images was provided by Fujifilm Medical Co. Ltd. The authors maintained full control over the study design, methods used, outcomes, and production of the written report.
